# On vision in birds: coordination of head-bobbing and gait stabilises vertical head position in quail

**DOI:** 10.1186/1742-9994-11-27

**Published:** 2014-03-25

**Authors:** John A Nyakatura, Emanuel Andrada

**Affiliations:** 1 Institut für Spezielle Zoologie und Evolutionsbiologie mit Phyletischem Museum, Friedrich-Schiller-Universität, Erbertstraße 1, 07743 Jena, Germany; 2 Science of Motion, Institut für Sportwissenschaft, Friedrich-Schiller-Universität, Seidelstraße 20, 07743 Jena, Germany

**Keywords:** *Coturnix*, X-ray motion analysis, Center of mass mechanics, Head-bobbing, Vision, Locomotion

## Abstract

**Introduction:**

Head-bobbing in birds is a conspicuous behaviour related to vision comprising a hold phase and a thrust phase. The timing of these phases has been shown in many birds, including quail, to be coordinated with footfall during locomotion. We were interested in the biomechanics behind this phenomenon. During terrestrial locomotion in birds, the trunk is subjected to gait-specific vertical oscillations. Without compensation, these vertical oscillations conflict with the demands of vision (i.e., a vertically stable head position). We tested the hypothesis that the coordination between head-bobbing and trunk movement is a means of reconciling the conflicting demands of vision and locomotion which should thus vary according to gait.

**Results:**

Significant differences in the timing of head-bobbing were found between gaits. The thrust phase was initiated just prior to the double support phase in walking (vaulting) trials, whereas in running (bouncing) trials, thrust started around midstance. Altering the timing of head-trunk-coordination in simulations showed that the timing naturally favoured by birds minimizes the vertical displacement of the head. When using a bouncing gait the timing of head bobbing had a compensatory effect on the fluctuation of the potential energy of the bird’s centre of mass.

**Conclusion:**

The results are consistent with expectations based on the vertical trunk fluctuations observed in biomechanical models of vaulting and bouncing locomotion. The timing of the head-bobbing behaviour naturally favoured by quail benefits vision during vaulting and bouncing gaits and potentially helps reducing the mechanical cost associated with head bobbing when using a bouncing gait.

## Introduction

**Figure 1:**
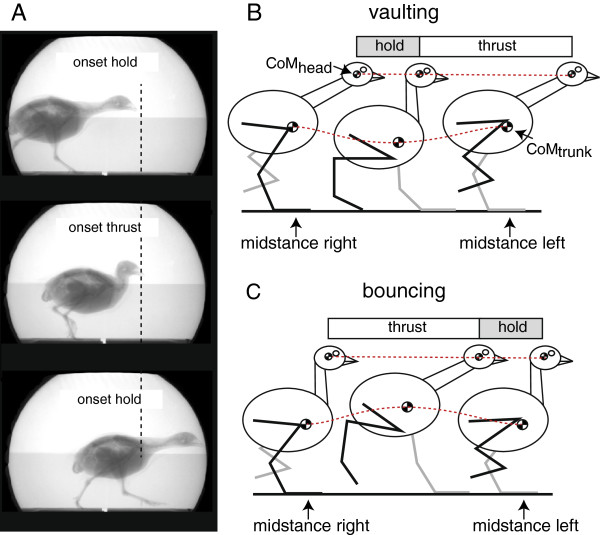
**Head-bobbing in quail. A**: Still images from a representative x-ray sequence of a quail during grounded running. During the hold phase the head is held in a fixed position relative to space, while the rest of the body continues to move forward. During the thrust phase the head is moved forward rapidly relative to the body. **B**: Coordination between head-bobbing and gait during a trial with vaulting mechanics as a means of minimizing vertical head displacement during locomotion. The trunk is subjected to specific rhythmic vertical displacements (fluctuations of *E*_*p*_) depending on the gait. We hypothesized that the onset of the thrust phase would occur around the event where the *E*_*p*_ of the *CoM*_*trunk*_ was at a minimum since the extension of the neck may function to reduce the vertical displacement of the head (eyes) and is thus potentially advantageous to vision. In this view, coordination between head-bobbing and gait during a trial with bouncing mechanics **(C)** would thus require a different timing of head-trunk coordination.

Head-bobbing is primarily an optokinetic phenomenon occurring during terrestrial locomotion in many birds [1-7]. It comprises a hold phase (head position is fixed relative to the environment while the body continues to move forward) and a thrust phase (the head is thrust forward relative to the body; Figure 1A). This rapid forward movement of the head is believed to permit depth perception *via* motion parallax in a visual field with limited overlap between the left and the right eye [3,8,9]. Because the magnitude of the effect of motion parallax depends on the extent of head movement [10,11], depth cues are maximized and the largest difference in the apparent position of an object perceived if the eyes are moved in the direction of movement parallel to the ground. This is because with such a trajectory head displacement is maximized relative to the direction movement. The hold phase allows more detail to be discerned by the stabilized retina [1,7,12,13]. It is long known for many species of bird including quail that head-bobbing behaviour is usually coordinated with footfall [1,5,14-18]. However, the functional significance of this coordinated movement is uncertain. Abourachid and colleagues proposed that the trunk motions might be a trigger for head stabilisation [18]. In this view, coordination of head-bobbing and footfall might represent a side effect of a coordination of trunk motion and head motion, because trunk motions are inextricably related to footfalls [18-20]. We here follow this idea and study head stability of head-bobbing quail in relation to gait.

The vertical displacements to which the body is subjected during locomotion are usually reflected by energy fluctuations in the body’s centre of mass (CoM), which themselves vary significantly between gaits [21-23]. In a vaulting gait, the body’s potential energy (*E*_*p*_) is at a minimum around double support. In bouncing gaits, *E*_*p*_ is at a minimum around midstance. Therefore, a vertically stable head position needed for vision during head-bobbing potentially conflicts with the vertical oscillations of the body’s CoM during terrestrial locomotion.

We hypothesised the timed coordination between head-bobbing and gait to be a means of mitigating disadvantageous effects both behaviours exert on each other. Specifically, we hypothesised the timing of head-bobbing to limit the vertical oscillation of the head and expected the timing to reflect the type of gait used (Figure 1B, C). Theoretically, the thrust phase should start when the trunk is about to move up (i.e., when *E*_*p*_ is at a local minimum). In a bouncing gait, then, the onset of thrust was expected around midstance, while in a vaulting gait it was expected at the beginning of the double support phase. We analysed head-bobbing in unrestrained vaulting and bouncing gaits in the common quail, conducting x-ray motion analyses to study body segment kinematics. Additionally, we simulated trials in which head-bobbing did not occur and trials in which the timing of head-bobbing was inversed in order to study the effects of modified head-bobbing behaviour. In representative vaulting and bouncing trials, we simulated trials in which we stepwise shifted the onset of thrust to test whether quail optimize the timing of head-bobbing in line with our hypothesis.

## Materials and methods

Eight common quails (Galliformes: *Coturnix coturnix* L. 1758) weighing between 180 g and 247 g (mean ± standard deviation: 207.4 ± 22.3 g, Table 1) were obtained from a local breeder and housed in four spacious cages at the Institut für Spezielle Zoologie und Evolutionsbiologie in Jena, Germany. Animals were kept and all experiments were conducted in strict adherence to the regulations of the committee for animal welfare of the state of Thuringia (registration number: 02-047/10). For this study we used a dataset (*x, y, z* coordinates of skeletal landmarks of quail during unrestrained locomotion, see below) that has been previously used [24,25]. However, the present study required new analyses of the available data. This multiple use of raw data allowed us to limit unnecessary exposition of the study subjects to potentially harmful radiation.

**Table 1:** Body mass and number of trials that met criteria for analysis of participating quail individuals

**Animal no.**	**Mass**	**Trials**
1	230	1
2	210	6
3	190	12
4	202	15
5	190	4
6	247	6
7	210	4
8	180	1

### Determination of the CoM

**Figure 2:**
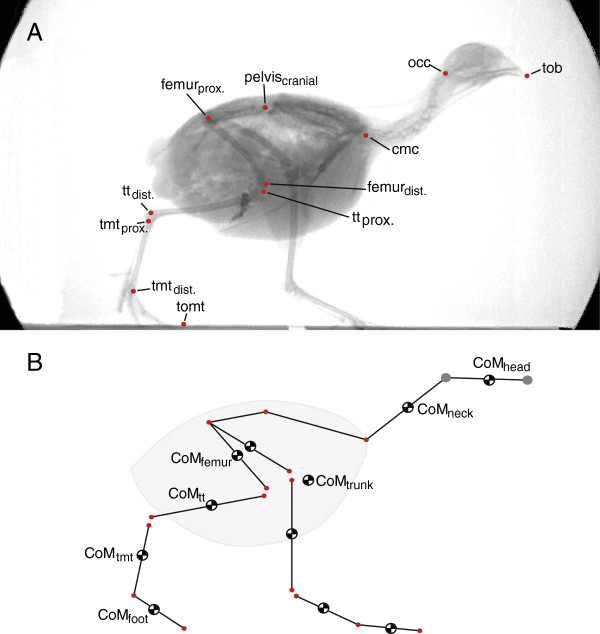
**Kinematic analysis and the kinematic model used for the calculation of the body’s CoM (center of mass). A**: Lateral x-ray projection to show the skeletal landmarks digitized. Landmarks were identified and digitized non-automatically. Both hindlimbs were digitized though landmarks for one limb are only shown here. **B**: Stick figure representation of the kinematic model. The mass and precise CoM position (using a pendulum method) of each segment was determined [23]. The instantaneous position of the body’s CoM was calculated by combining the kinematic data, the mass and inertial properties. Please note that CoM_trunk_ and the CoM of the whole body are not identical. In simulations only the timing of the head landmarks (grey) were modified with respect to the experimental data (red) of the remaining landmarks. Tob: tip of beak; occ: occiput; cmc: caudal-most cervical vertebra; tt: tibiotarsus; tmt: tarsometatarsus; tomt: tip of middle toe.

To determine the instantaneous position of the CoM over the course of the step, individual segments (toes, tarsometatarsus, tibiotarsus, femur, trunk, neck and head) of two quails (230 g and 247 g, respectively) were weighed and the CoM of each segment determined using a previously published pendulum procedure and custom MATLAB scripts [23]. The segments were defined using the landmarks of the x-ray motion analysis (XMA see below, Figure 2). The mass and inertial properties of each segment are published elsewhere [24]. The head and neck combined make up 10.5% of a quail’s body mass, whereas each hindlimb makes up 8.5% and the trunk including the wings accounts for 73.5% [24]. The positions of the CoM of individual segments were linearly scaled relative to segment length to account for size differences between subjects. We calculated the instantaneous position of the birds’ CoM from kinematic data (XMA of the limbs, additional kinematic data pertaining to digitized head, neck, and torso landmarks; see below) and the position of the CoM of each element. The CoM position derived using this method corresponds to the position derived by integrating ground reaction force data [24,26]. Note that the instantaneous position of the CoM was determined for both, the original experimental data and the simulated trials (see below).

### X-ray motion analysis (XMA)

On recording days, 4 individuals were taken to the x-ray motion analysis lab for experiments. At least one day of rest was given to each bird between recording days. All animals were allowed to rest in transport boxes for at least an hour prior to experiments and between sets of trials to limit stress. No more than 10 consecutive trials were conducted with each individual regardless of whether the trials could be used for further analyses. Each trial involved a subject walking or running (in most cases spontaneously) along a 3 m track that passed two orthogonal x-ray beams at its own preferred speed. Synchronous x-ray videos were recorded from the ventral projection and the lateral projection using a fully digital biplane high-speed x-ray video unit (specifics of the unit are detailed in [27]) that captured about 30 cm of the track. The x-ray system operated at 40 kV and 53 mA and recorded images of 1.536×1.024 pixels at 1 kHz. No additional shutter was necessary to further reduce motion blur. The inherent distortion in the x-ray image was corrected using a recorded image of a metal grid with known geometry and comparing the spatial geometry of the image and the grid to define a projection rule [28]. All images from the analysed trials were subsequently corrected using this rule (MATLAB scripts available free of charge from http://www.xromm.org).

A calibration object into which metal beads were inserted at known distances was also recorded in both projections at the end of each recording day. Direct linear transformation (DLT) was performed using SimiMotion 3D (Simi Reality Systems, Unterschleißheim, Germany) to 3D calibrate the recording space. Undistorted and calibrated x-ray videos were used to obtain 3D coordinates of skeletal landmarks, usually a proximal and a distal landmark for each segment (Figure 2). Landmarks were digitized on at least every tenth frame and then spline interpolated over the entire image sequence. Close to lift-off and touch-down we digitized every third frame to precisely characterise these important events of the stride cycle. The XMA of individual trials thus resulted in a set of *x,y,z* coordinates for each landmark over time. Using connecting lines between landmarks belonging to a segment, the kinematics of a trial can be visualized as a moving stick-figure for example in MATLAB (see Additional file 1).

To address the specific question of this study, namely the coordination between fore-aft head movements relative to the vertical movements of the body’s CoM, we restricted our analysis to the sagittal plane kinematics and CoM energy patterns. Medio-lateral amplitudes of the movements of the head’s and the body’s CoM were found to be small during vaulting trials (0.23 ± 0.1 cm and 0.22 ± 0.12 cm, respectively) and bouncing trials (0.21 ± 0.12 cm and 0.22 ± 0.11 cm, respectively) in the experimental data. Thus, the 3D data was projected to the sagittal plane (only *x* and *y* coordinates were used). A step was defined as lasting from midstance of one limb to midstance of the other limb. Subsequent stance phases needed to be completely within the field of view of the x-ray system. This approximately applied to just 10% of all trials. In addition, non-steady state trials with a horizontal speed deviation of more than 10% between two midstance events were discarded during later analysis. No runs with an aerial phase occurred that met our criteria. Altogether we analysed 49 trials (16 vaulting, 33 bouncing) with 98 strides (Table 1).

To differentiate vaulting from bouncing gaits we used % Congruity [29]. % Congruity is the percentage of overall frames making up a step which feature frame-to-frame changes in *E*_*p*_ and *E*_*k*_ of equal sign. Ideally, % Congruity would be 100% in a perfect bouncing (running) trial and 0% in a perfect vaulting (walking) trial. *E*_*p*_ was calculated as *E*_*p*_ = *mgy*, where *m* is mass, *g* is gravitational acceleration and *y* is the vertical oscillation of the CoM. *E*_*k*_ was calculated as $E_{k}=0.5m\left({\overset{˙}{x}}^{2}+{\overset{˙}{y}}^{2}\right)$, where $\overset{˙}{x}$ and $\overset{˙}{y}$ are the horizontal and vertical speed, respectively. However, gait transitions in birds have been demonstrated to be smooth [7,23,30,31]. We defined values of % Congruity <50% as vaulting-like and >50% as bouncing-like (Figure 1B). The birds bobbed their heads in all trials. The timing of the onset of thrust and hold phases and the timing of footfalls was determined from x-ray videos. The onset of the thrust phase was defined as the frame in which the relative horizontal distance between the tip of the beak and the caudal-most cervical vertebra exceeded by 2 mm its minimum value relative to the duration of the entire step.

### Simulations

All head bobbing data reported in this study stem from original experimental data and reflect the natural behaviour of the birds (all data from experimental data marked red in graphs). However, by manipulating the time functions of the coordinates of the head and neck in the experimental data of the original head bobbing trials, we also simulated trials without head-bobbing and those featuring inverse head-bobbing (all data from simulations marked grey in graphs). This was achieved using custom MATLAB scripts. In addition, we also manipulated the original onset of head-bobbing in our experimental data in some representative vaulting and bouncing trials to explore whether or not quail optimize the onset of thrust to minimize vertical head displacement. To this end, the phase shift between the time functions of the motion of the neck and the head relative to the trunk was varied stepwise (increments of 10% of stance duration). In simulated trials without head-bobbing, the positions of the neck and head were fixed in the kinematic model at the minimum value of the distance between the CoM of the head and the cranial pelvis landmark. To simulate inverse head-bobbing behaviour, the time functions of the motion of the neck and head segments relative to the trunk were inverted, resulting in a 180° phase shift compared to normal head-bobbing behaviour of the experimental data. The kinematic model used for these simulations is that from the XMA (experimental data) of each trial. Note that during simulations without head-bobbing, with inversed head-bobbing, or with shifted head-bobbing onset, the kinematics of the trunk and the extremities were never altered and are thus identical to the experimental data. In simulations just the motion of the head and neck relative to the trunk were manipulated. An animation of a grounded running gait featuring natural head-bobbing, no head-bobbing and inverse head bobbing can be found in the Additional file 1.

### Statistical analysis

Experimental data of the same gait from different subjects were pooled. All statistical analyses were performed in IBM SPSS Statistics 21 (IBM Corporation, Armonk, NY, USA). We used the Student’s *t*-test to compare the means of the onset of thrust and double support phase between the two gaits. We used a one-way ANOVA to compare the means of the various simulated trials, with post hoc pairwise Dunnett’s *t* tests to test for significant differences between the experimentally derived head-bobbing trial (experimental data) and the modified trials from the simulations. We also tested for differences between head displacement and the displacement of the CoM of the trunk in both experimentally derived and simulated trials. The significance level was set at *p* = 0.05 (marked by an asterisk in graphs), with higher significance marked by two asterisks (*p* < 0.01) and highest significance (*p* < 0.001) marked by three asterisks. Data is reported as mean ± standard deviation. To complement null hypothesis significance testing and to further differentiate the biological significance of the data, we also calculated effect size statistics (discussed in Nakagawa and Cuthill [32]). 95% confidence intervals (CI) are presented and a measure of standardized effect size (Cohen’s *d*) based on the difference between corresponding trials is provided. We use Cohen’s ‘conventional’ values as measures of a small, medium, and large effect (*d* = 0.2, 0.5, 0.8, respectively; [33]).

## Results

**Figure 3:**
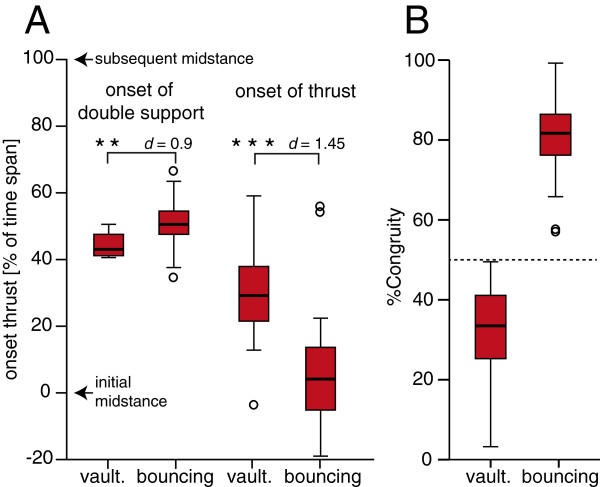
**Characteristics of the experimental data. A**: Onset of the double support phase and the thrust phase as a percentage of the time span between subsequent hindlimb midstance events. **B**: % Congruity was used to differentiate between trials with vaulting mechanics (< 50% congruity) and trials with bouncing mechanics (> 50% congruity). Boxes represent range between first and third quartiles (i.e.. 50% of the data), the line within the box represents the median and the length of each whisker corresponds to the lowest datum within 1.5 IQR (interquartile range) of the lower and 1.5 IQR of the upper quartile, respectively; outliers (more than 1.5 IQR) depicted as open circles; **: significant at the *p* = 0.01 level; ***: significant at *p* = 0.001 level; *d*: Cohen’s *d* (effect size).

The timing of thrust differed significantly between trials with vaulting mechanics (vaulting trials) and trials with bouncing mechanics (bouncing trials) (Figure 3). In vaulting trials, the onset of thrust largely overlapped with the onset of the double support phase. In bouncing trials, the onset of thrust occurred around midstance. The timing of head-bobbing (experimental data) resulted in significantly smaller vertical head displacements and thus a straighter trajectory for the head than in trials in which the timing of head-body-coordination was inversed (simulation data).

### Vertical head displacement

**Figure 4:**
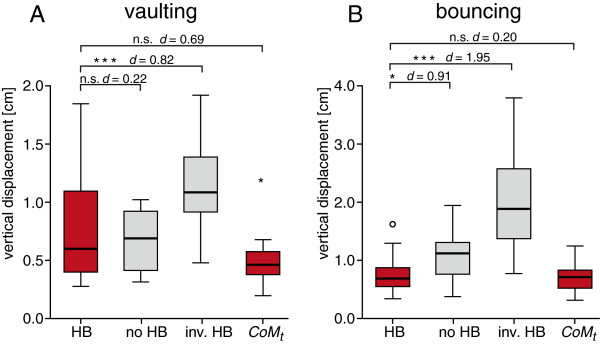
**Vertical displacement of the head in experimental trials (HB), simulated trials without head-bobbing (no HB), simulated trials in which the timing of head-bobbing was inversed (inv. HB) as well as vertical displacement of trunk’s center of mass (*CoM***_***t***_**). A**: vaulting, **B**: bouncing. Boxes represent range between first and third quartiles (i.e. 50% of the data), the line within the box represents the median and the length of each whisker corresponds to the lowest datum within 1.5 IQR (interquartile range) of the lower and 1.5 IQR of the upper quartile, respectively; outliers (more than 1.5 IQR) depicted as open circles; n.s.: not significant; *: significant at the *p* = 0.05 level; ***: significant at *p* = 0.001 level; *d*: Cohen’s *d* (effect size).

An ANOVA of vaulting trials revealed significant differences between experimental and simulated trials (*p* < 0.001) in regard of the vertical displacement of the head. Post hoc testing demonstrated that the vertical displacement of the head in vaulting trials (0.70 ± 0.51 cm) was not significantly larger (*p* = 0.123) than the vertical displacement of the CoM of the trunk (0.43 ± 0.25 cm), but with the effect size of the difference found to be medium (*d* = 0.69; Table 2; Figure 4A). Vertical head displacement in vaulting trials featuring natural head-bobbing did not differ significantly from vertical head displacement in simulated non-head-bobbing trials (0.61 ± 0.28 cm; *p* = 0.404), but effect size statistics still revealed a small effect (*d* = 0.22). However, natural head-bobbing (experimental data) resulted in significantly smaller vertical head displacements and a straighter trajectory of the head than inverse head-trunk-coordination with a large effect size (1.08 ± 0.41 cm; *p* < 0.001; *d* = 0.82).

**Table 2:** Post hoc comparison and effect size statistics of original trials (head-bobbing; HB), simulated trials without head-bobbing (no HB), and simulated trials in which the timing of head-bobbing was inversed (inv. HB)

	**Mean difference**	**95% CI of mean difference**	**Significance (Dunnett’s** ***t*)**	**Effect size (Cohen’s** ***d*)**
*Vertical displacement of the head*				
Vaulting				
No HB *vs.* HB	–0.9214 cm	± 0.3319	0.842	0.22
Inv. HB *vs.* HB	0.3787 cm	± 0.3319	0.021*	0.82
CoM *vs.* HB	–0.2762 cm	± 0.3320	0.123	0.69
Bouncing				
No HB *vs.* HB	0.3389 cm	± 0.3131	0.03*	0.91
Inv. HB *vs.* HB	1.3145 cm	± 0.3131	< 0.001***	1.95
CoM *vs.* HB	0.5885 cm	± 0.3132	0.944	0.20
				
*Amplitude of E*_*p*_*fluctuation*				
Vaulting				
No HB *vs.* HB	0.3639 mJ	± 3.7318	0.964	0.08
Inv. HB *vs.* HB	0.8127 mJ	± 3.7319	0.837	0.18
Bouncing				
No HB *vs.* HB	0.9257 mJ	± 2.1613	0.530	0.24
Inv. HB *vs.* HB	2.8846 mJ	± 2.1614	0.096	0.48
				
*Amplitude of E*_*k*_*fluctuation*				
Vaulting				
No HB vs. HB	–1.9423 mJ	± 4.3011	0.486	0.35
Inv. HB vs. HB	–3.4037 mJ	± 4.3012	0.137	0.67
Bouncing				
No HB vs. HB	–2.2789 mJ	± 2.9916	0.159	0.38
Inv. HB vs. HB	–2.0391 mJ	± 2.9915	0.223	0.36

*: significant at the p = 0.05 level; ***: significant at the p = 0.001 level.

In bouncing trials, we found highly significant differences between groups (*p* < 0.001; Figure 4B). Post hoc tests showed that the vertical displacement of the head was significantly smaller in experimental trials (0.65 ± 0.32 cm) than in simulated trials without head-bobbing (0.99 ± 0.42 cm; *p* = 0.021) and also smaller than in simulated trials featuring inverse head-bobbing (1.96 ± 0.9 cm; *p* < 0.001). In both cases, the effect size clearly exceeded the conventional value of a large effect (*d* = 0.91 and *d* = 1.95, respectively). Vertical displacement of the head in bouncing trials did not differ from the vertical displacement of the CoM of the trunk (0.59 ± 0.25 cm; *p* = 0.944) in experimental trials, though an effect was still found (albeit a marginal one: *d* = 0.2).

**Figure 5:**
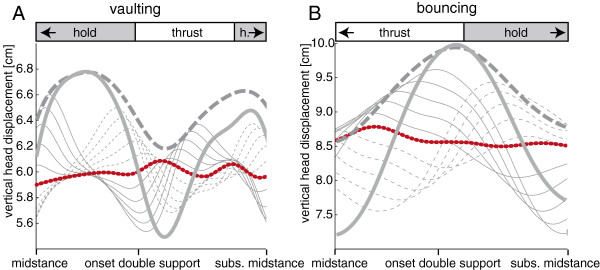
**Representative traces of the vertical displacement of the head from midstance to subsequent midstance for a vaulting trial (A) and a bouncing trial (B).** Dotted red lines represent original trials with head bobbing, thick dashed grey lines represent simulated trials without head-bobbing, and thick solid grey lines represent trials in which head-bobbing was inversed. Thin grey lines represent stepwise modifications of the onset of thrust. Each step was an increase (solid lines) or decrease (dashed lines) by 10% of stride duration. In the vaulting trial the quail moved at 0.15 m/s and % Congruity was 24.7. In the bouncing trial the quail moved at 0.55 m/s and % Congruity was 93.8. Note that in the vaulting trial the initial hold phase begins slightly before the initial midstance and the subsequent hold phase continues beyond the subsequent midstance. In the bouncing trial the thrust phase begins slightly before the initial midstance and the hold phase continues slightly beyond the subsequent midstance. In both cases, the timing of the thrust phase (experimental data) results in smaller vertical head displacement in comparison to simulated head-trunk-coordination patterns in the respective gait.

Stepwise shifts in the onset of thrust in simulations demonstrated that the timing naturally favoured by quail (experimental data) results in smaller vertical displacement of the head in both gaits (Figure 5).

### Energy fluctuations of the CoM

**Figure 6:**
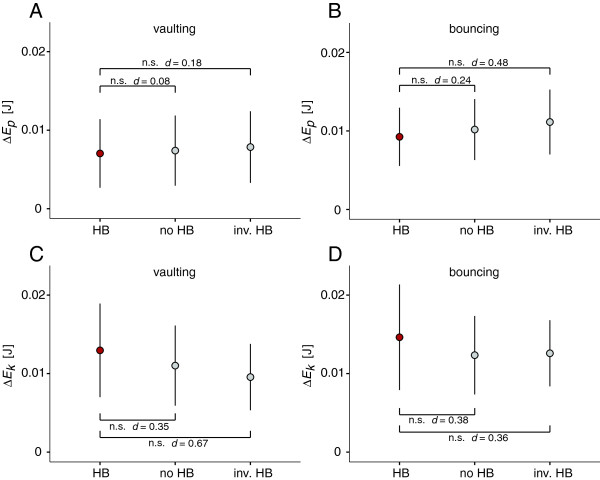
**Mean amplitude (± s.d.) of*E***_***p***_**and*E***_***k***_**fluctuation (*ΔE***_***p***_**and*ΔE***_***k***_**) in experimental trials (HB), simulated trials without head-bobbing (no HB) and simulated trials with inversely timed head-bobbing behavior (inv. HB). A**: *ΔE*_*p*_ in vaulting trials; **B**: *ΔE*_*p*_ in bouncing trials; **C**: *ΔE*_*k*_ in vaulting trials; **D**: *ΔE*_*k*_ in bouncing trials.

Modified head-bobbing behaviour also influenced the amplitude of *E*_*p*_ and *E*_*k*_ fluctuations in the CoM. However, this influence is only reflected in effect size statistics and only for bouncing trials (Table 2; Figure 6A). In regard of *E*_*p*_ fluctuations, ANOVAs for both vaulting and bouncing trials were non-significant (*p* = 0.883 and *p* = 0.153, respectively). In experimental vaulting trials, fluctuations in *E*_*p*_ (0.007 ± 0.0044 J) did not differ significantly from simulated vaulting trials which did not feature head-bobbing (0.0074 ± 0.0044 J; *p* = 0.964) or from simulated trials in which the timing of head-bobbing was inversed (0.0078 ± 0.0045 J; *p* = 0.837) – as revealed by post hoc tests. Effect sizes were also minimal (Table 2).

In contrast, and despite similar values overall, bouncing trials featuring normal (i.e., experimental) head-bobbing behaviour always yielded smaller *E*_*p*_ fluctuation values (0.0093 ± 0.0037 J) than simulated trials without head-bobbing (0.0102 ± 0.0039 J) and simulated trials in which the timing of head-bobbing was inversed (0.0111 ± 0.0041 J; Figure 6B). Therefore, even though differences were not significant for either simulated head bobbing strategy in bouncing gaits (*p* = 0.53 and *p* = 0.096, respectively), small effects were found when experimental head-bobbing trials were compared with simulated trials without head-bobbing and simulated trials in which the timing of head-bobbing was inversed (Table 2).

In regard of *E*_*k*_ fluctuations, ANOVAs for vaulting and bouncing trials were non-significant as well (*p* = 0.204 and *p* = 0.176, respectively). For vaulting trials post-hoc testing showed that neither simulated trials without head bobbing (0.011 ± 0.0051 J, *p* = 0.486) nor simulated trials with inversed timing of head bobbing (0.0096 ± 0.0042 J, *p* = 0.137) differed significantly from the experimental trials (0.0123 ± 0.006 J; Figure 6C). However, fluctuations of *E*_*k*_ tended to be smaller in simulated trials reflected in small and medium effect sizes (Table 2).

*E*_*k*_ fluctuations of bouncing trials again did not differ significantly from experimental data (0.0146 ± 0.0067 J) in simulated trials without head bobbing (0.0123 ± 0.005 J, *p* = 0.159) and simulated trials with inversed timing of head bobbing (0.0126 ± 0.0042 J, *p* = 0.223; Figure 6D). Nevertheless, effect size statistics revealed small effects (Table 2).

## Discussion

The coordination between head-bobbing and hindlimb movement has been documented for numerous species of bird [1,5,14-17]. Fujita [16] established that the timing and amplitude of head-bobbing is correlated to stride length and proposed that the behaviour has a displacing effect on the animal’s CoM which is advantageous to locomotor stability—an idea first put forward by Dagg [34]. However, the displacing effect on the CoM was actually found to be very small [16,35,36]. Birds do not bob their heads when walking or running on a treadmill [3] or when blindfolded [1]. The importance of head-bobbing for dynamic stability during terrestrial locomotion therefore seems to be negligible [9].

It is important to note that a recent study on tinamous questions a timed coordination of footfall and head bobbing behaviour [19]. There certainly is variation of the timing of this coordination *within* data of the gaits in quail, too (Figure 3). However, we here demonstrate that the timing of head-bobbing is coordinated with fluctuations in the *E*_*p*_ of the birds’ trunk which is fundamentally different in the two mechanically defined gaits. This specific hypothesis was not tested in the previous study on tinamous [19] and therefore results of this previous study are not mutually exclusive to the results presented here. As hypothesised (Figure 2), the timing of head-bobbing in quail differs significantly *between* vaulting and bouncing gaits, with thrusts occurring immediately prior to the double support phase in vaulting trials. This finding is consistent with the idea that the timing of head-bobbing – not the behaviour itself – as a subordinate phenomenon functions to mitigate the conflict between the need for vertically stable eye movement and the vertical fluctuation of the trunk. This result was clearest in trials with bouncing mechanics. However, an alternative benefit of the timing between head and trunk movements observed in this study could be an advantage for re-directing the CoM trajectory after it reaches the minimum in *E*_*p*_ by providing acceleration of the CoM. This event occurs during the double contact phase in vaulting trials, whereas it occurs around midstance in bouncing trials (Figure 1B, C).

When compared to simulated trials in which the timing of head-bobbing was inversed, the timing naturally favoured by the birds had significantly smaller vertical excursions of the head. Jiménez Ortega and colleagues [9] demonstrated in pigeons that shape discrimination during the thrust phase is as good as during the hold phase and concluded that unlike previously supposed [8,37], vision is not suppressed during the thrust phase [9]. Optic flow is more pronounced in the lateral field of view than in the frontal field [9] and more prone to suffer from translational and rotational perturbations [12]. The vertical stabilisation of the eye documented here, which is effected by gait-specific head-trunk coordination during terrestrial locomotion, is therefore advantageous to vision. Similar vertical stabilization of the eyes is also achieved in herons during perch perturbations [38].

The effects observed in our study were more marked in trials involving bouncing gaits. This can be attributed to the fact that in grounded running, the preferred gait of ground-dwelling birds (see [22,24,25,39]), the vertical fluctuations of the trunk are large (e.g., [7,22-24]), making the benefits of head stabilising mechanisms particularly visible. At the highest speeds many birds abandon head-bobbing behaviour altogether [17,19].

In addition to the smaller vertical excursions of the head, it was observed that the natural timing of head-bobbing has a compensatory effect on the *E*_*p*_ fluctuations in the birds’ CoM during bouncing gaits. When comparing experimental bouncing trials with trials that simulated inversed head-bobbing we found nearly a medium-sized effect. Thus, this limitation of *E*_*p*_ fluctuation potentially constitutes an energy-saving mechanism since all positive increments in *E*_*p*_ are costly in terms of metabolic energy (see, for example [40]). That is, because head bobbing is primarily necessary for vision and this behaviour is associated with mechanical costs. These costs arise from the additional fore-aft accelerations of the CoM that increase positive increments of *E*_*k*_ (see higher *E*_*k*_ fluctuations in the experimental data than in simulated data with modified timing of head bobbing in Figure 6C, D). The here documented timing of head bobbing during bouncing trials has the effect of limiting the positive increments of *E*_*p*_ and is thus potentially decreasing, albeit to a rather small extent, the additional cost connected to head-bobbing. Here too, it is the timing and coordination of head-bobbing with gait specific trunk vertical motion which provides the benefit, not the behaviour *per se*.

The data of this study supports the notion that head-bobbing is coordinated with gait in quail to mitigate the adverse effects of locomotion on vision. Given the overall similarity between head-bobbing in quail and the same behaviour in other birds, these results might apply to many taxa of head-bobbing bird.

## Competing interests

The authors declare that they have no competing interests.

## Authors’ contributions

JAN and EA conceived of the study and conducted all experiments. JAN analysed the kinematic data and performed the statistical analysis. EA did the simulations. JAN drafted the manuscript. Both authors read and approved the final version of the manuscript.

## Supplementary Material

Additional file 1 A short movie of a head-bobbing quail during unrestrained grounded running (lateral and ventral projections) and of our kinematic model with original and modified head-bobbing behaviour. Click here for file
